# A Plasmonic Infrared Multiple-Channel Filter Based on Gold Composite Nanocavities Metasurface

**DOI:** 10.3390/nano11071824

**Published:** 2021-07-14

**Authors:** Jialin Zhang, Xuanyi Yu, Jingxin Dong, Weiji Yang, Shuang Liu, Chongyang Shen, Jiacheng Duan, Xiaoxu Deng

**Affiliations:** 1 State Key Laboratory of Advanced Optical Communication Systems and Networks, Key Laboratory for Laser Plasmas (Ministry of Education), School of Physics and Astronomy, Shanghai Jiao Tong University, Shanghai 200240, China; dorismalfoy@sjtu.edu.cn (J.Z.); yxy1593725@sjtu.edu.cn (X.Y.); yangweiji@sjtu.edu.cn (W.Y.); 503313461@sjtu.edu.cn (S.L.); cy_shen@sjtu.edu.cn (C.S.); duanjiacheng@sjtu.edu.cn (J.D.); 2 School of Electronic Information and Electrical Engineering, Shanghai Jiao Tong University, Shanghai 200240, China; jx_dong@sjtu.edu.cn

**Keywords:** metasurface, nanocavities, plasmonic filter

## Abstract

A plasmonic near-infrared multiple-channel filter is numerically and experimentally investigated based on a gold periodic composite nanocavities metasurface. By the interference among different excited plasmonic modes on the metasurface, the multipeak extraordinary optical transmission (EOT) phenomenon is induced and utilized to realize multiple-channel filtering. Investigated from the simulated transmission spectrum of the metasurface, the positions and intensity of transmission peaks are tuned by the geometrical parameters of the metasurface and environmental refractive index. The fabricated metasurface approached transmission peaks at 1128 nm, 1245 nm, and 1362 nm, functioning as a three-passbands filter. With advantages of brief single-layer fabrication and multi-frequency selectivity, the proposed plasmonic filter has potential possibilities of integration in nano-photonic switching, detecting and biological sensing systems.

## 1. Introduction

Metamaterial, especially its two-dimensional equivalents, i.e., metasurface, has aroused widespread attention due to its splendid electromagnetic wave manipulations properties, such as beam steering [1], radiation patterns reconfiguration [2], and nearfield transformation [3]. Plasmonic metamaterials based on metal nanocavities, exhibiting notable optical properties, including extraordinary optical transmission (EOT) [4], negative refractive index [5], and enhancement of nonlinear effect [6,7], has been an active research field in past decades, which provide great prospects of the application in sensing [8,9,10], plasmonic color filtering [11,12,13,14], and subdiffractive imaging [15], etc. Metallic nanocavity concentrates optical energy to deep subwavelength regions by the excitation of surface plasmons, inducing confinement of electromagnetic fields with frequency-selective features [16,17]. Periodic nanometallic cavities arrays in metamaterials support plasmons near-field coupling among cavities [18], leading to collective resonances such as the EOT phenomenon at selectable wavelength. Further on, the composite cavities structure is introduced into the metamaterial [19,20,21], which generates an interaction giving rise to plasmonic hybridization and strong optical field coupling among spectrally-overlapped modes, resulting in the additive spectral response of metamaterial [22,23,24,25]. For example, a polarization-insensitive NIR filter is presently based on asymmetry metallic elliptical and circle nanocavities array metasurface [26], exhibiting 79 nm narrow linewidth generated by a Fano resonance. The invertible plasmonic spin-Hall effect at the nanoscale is achieved by breaking the spin degeneracy through the interference among the different plasmon resonances in the U-shaped cavity metasurface [27]. An absorptive-type metasurface color filter is realized through truncated-cone hyperbolic metamaterial absorbers consisting of several layers of metal-dielectric films with tapered angles [28]. Hence, with increasingly matured nanofabrication, the realization of multi-wavelength and high-efficiency sub-wavelength optical manipulation with a single-layer metasurface is worth investigating.

In this paper, a multi-channel infrared plasmonic filter based on a gold periodic composite nanocavities metasurface is numerically and experimentally investigated. Plasmonic modes excited in single horizontal nanocavity structure and double vertical nanocavities structure are simulated by the finite-difference time-domain (FDTD) solutions, of which the interference induces multipeak extraordinary optical transmission phenomenon utilized in a plasmonic multi-frequency selective filter. By numerically analyzing the transmission spectrum of the metasurface, the transmission coefficient, operating channels, and linewidths of the plasmonic filter are tuned by cavities’ geometric parameters and metasurface periods, especially the operating channels, which are also tuned by the environment refractive index. The maximum transmission intensity and narrowest FWHM (full width at the half-maximum) of the EOT peaks are optimized in the simulation as 73% and 8 nm, respectively. The gold composite nanocavities metasurface was fabricated by Electron-Beam Lithography and Ion Beam Etching technique. The transmission spectrum of the fabricated metasurface was measured by a Fourier-transform infrared spectrometer with three passbands at wavelength 1128 nm, 1245 nm, and 1362 nm, which has good accordance in spectral positions with the simulation. Due to the advantages of multi-frequency selectivity and the single-layer simple fabrication process, the plasmonic filter based on the composite nanocavities metasurface has the potential to be a spatial component in high precision optical systems such as biosensors and photodetection technologies.

## 2. Materials and Methods

**Figure 1:**
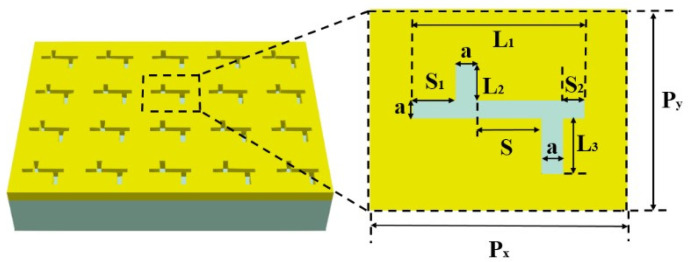
A schematic diagram of the periodic composite nanocavities metasurface.

The schematic of a periodic composite nanocavities metasurface on SiO_2_ substrate is shown in Figure 1. The unit cell of the metasurface has composite gold nanocavities, including one horizontal cavity connected with two vertical cavities on the opposite side. The length of the horizontal cavity is L1 = 800 nm, and the lengths of vertical nanocavities are L2 = 200 nm and L3 = 300 nm. Two vertical cavities are pointing upwards and downwards with a distance S1 = 200 nm and S2 = 100 nm to the left and right end of the horizontal cavity, respectively. The distance between two vertical cavities is S = 300 nm. The width of all three composite nanocavities is a = 100 nm. The periods of the metasurface are Px = 1200 nm and Py = 1100 nm. The thickness of the metasurface is h = 225 nm. The environment refractive index n_e_ = 1. The refractive index of SiO_2_ substrate is *n* = 1.45. The transmission spectra and the electric field distributions of the metasurface are simulated by the three-dimensional FDTD solution. In the 3D FDTD simulations, the Y-polarized light (900 nm–1500 nm) is illuminated along the Z-axis. The dielectric parameter of Au in the FDTD simulation is set as the Palik model. A semi-infinite SiO_2_ substrate is added below the metasurface. Periodic boundary conditions (PBCs) are used in the x- and y-directions, and perfectly matched layers (PMLs) are applied in the z-direction. The minimum mesh step is set as 0.25 nm.

In the fabrication process, the 225 nm-thick gold film was deposited on the SiO_2_ substrate by the Electron Beam evaporator (Denton Electron Beam Evaporator, Shanghai, China). The thickness of the SiO_2_ substrate is 1 mm. The composite nanocavities structure was then fabricated on the gold film by Electron-beam lithography (EBL, Vistec EBPG-5200+, Shanghai, China) process with AR-P 6200 polymethylmethacrylate (PMMA) positive photoresist of 200 nm thickness. The structure was then developed and fixed using MIBK and IPA. Ion Beam Etching (IBE, Ion Beam Etching System, Shanghai, China) technique was utilized to remove the photoresist using Ar gas. The size of the fabricated area is 1.2 mm × 1.1 mm. The geometrical parameters were measured in the scanning electron microscopy (SEM, Zeiss Ultra Plus Field Emission Scanning Electron Microscope, Shanghai, China) image. The transmission spectrum of the fabricated metasurface is detected by a Fourier-transform infrared spectrometer (Fourier-transform infrared spectrometer, Shanghai, China) with a linear-polarized source.

## 3. Results and Discussion

### 3.1. Simulated Results

**Figure 2:**
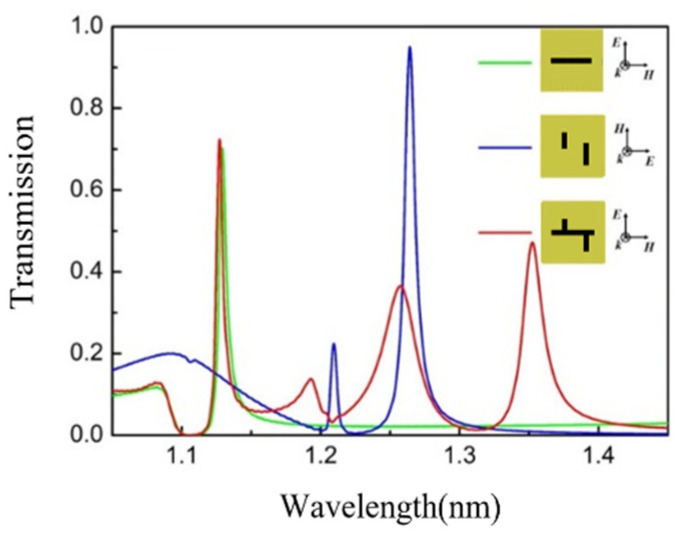
Normalized transmission spectrum (T) of the single nanocavity metasurface, double nanocavities metasurface, and composite nanocavities metasurface.

**Figure 3:**
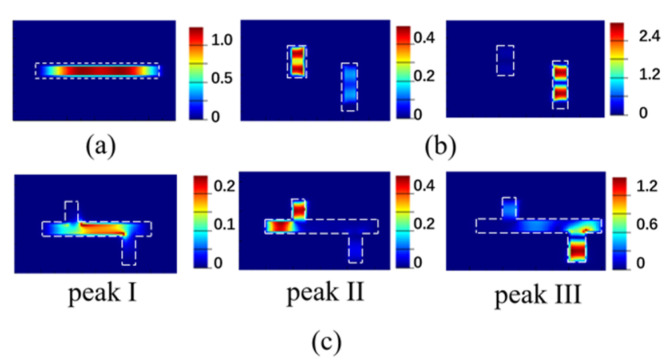
Simulated electric field distributions at the corresponding transmission peaks in cross-sections of (**a**) the single-horizontal nanocavity structure, (**b**) the double-vertical nanocavities structure, and (**c**) the composite nanocavities structure.

**Figure 4:**
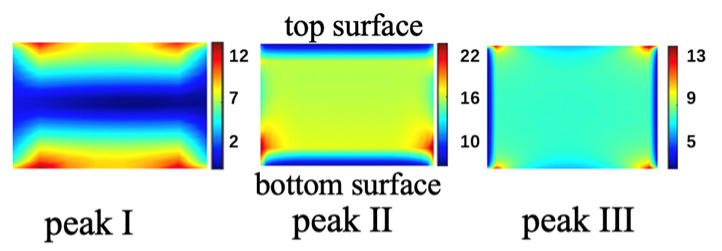
Simulated electric field distributions in cross-sections of x-z plane at three transmission peaks of the composite nanocavities structure.

The transmission spectrum of the single-horizontal nanocavity metasurface, the double-vertical nanocavities metasurface, and the composite nanocavities metasurface are simulated by the FDTD solution. Under a Y-polarized incident field, the single-horizontal cavity metasurface obtains an EOT peak of 82% at 1132 nm, shown as the green curve in Figure 2. At the 1132 nm transmission peak, the electric field is enhanced within the horizontal cavity, as shown in Figure 3a, which is induced by the excited surface plasmon polaritons (SPPs). Consequently, the single-horizontal cavity structure serves as the basic mode structure. The simulated transmission spectrum (blue curve in Figure 2) of double-vertical cavities metasurface under the X-polarization incident has two transmission peaks at wavelengths 1205 nm and 1280 nm of 23% and 92% transmittance, respectively, which has no transmission peak at this waveband with Y-polarization incident light. Similarly, the electric field is confined within the left and right vertical cavity at transmission peaks, respectively, as shown in Figure 3b. The transmission spectrum of composite cavities metasurface with horizontal and vertical cavities under Y-polarized incident is simulated, shown as the red curve in Figure 2, in which three EOT peaks, namely peak I, II, and III, appear at 1126 nm, 1255 nm, and 1350 nm with 73%, 36%, and 43% transmittances, respectively. At transmission peak I, the electric field is enhanced within the horizontal cavity, shown in Figure 3c, which is directly motived by the basic mode before. Meanwhile, newly generated transmission peaks II and III induced from the interaction between different modes, where the electric field is enhanced within the L-shaped junctions of the vertical cavities and horizontal cavity, which are red-shifted in peak position comparing with the plasmonic modes in vertical cavities. Plasmonic hybridization is generated in composite nanocavities, resulting in additive transmission spectral response. At three transmission peaks, the near-field electrical field is confined within the nanocavities due to the SPPs resonance, as shown in Figure 4. The confinement of the near-field electrical field is also influenced by the surrounding environment [29]. The multipeak extraordinary optical transmission phenomenon implemented by the composite nanocavities metasurface is utilized in multi-channel infrared plasmonic filtering.

**Figure 5:**
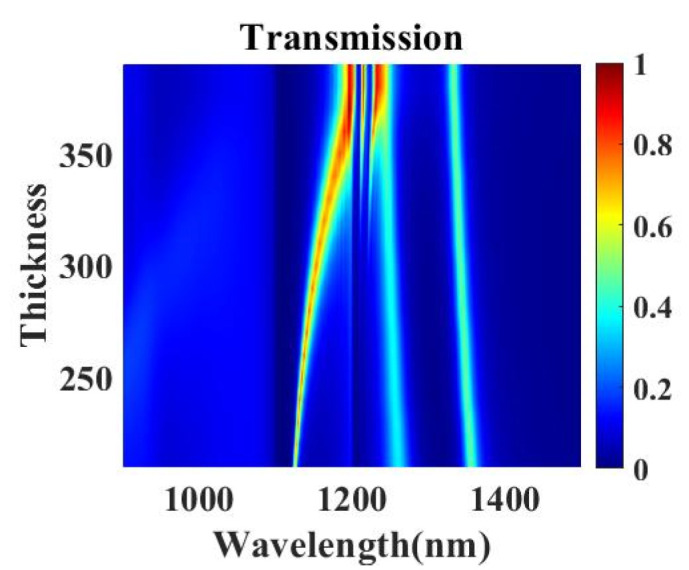
Simulated transmission spectra of the composite nanocavities metasurface with different Au film thicknesses h.

To meet various practical demands, the impact on the transmission spectra with several geometrical parameters of the metasurface is investigated, offering different parameter selection strategies of choosing transmission intensity, frequency positions and spacing of transmission peaks together or individually. The influence of Au film thickness on the transmission spectrum of composite nanocavities metasurface is investigated by FDTD. With Au film thickness h increasing from 200 to 400 nm, EOT peak I presents an obvious red shift and a higher transmittance with the broadening of FWHM, and Peak II and III are just slightly blue-shifted in Figure 5, which leads to peak I and peak II overlapped when h is over 350 nm. The increase in Au thickness causes more electric fields confined inside the horizontal cavity and more material dissipation, leading to the variation of transmission peak I both in FWHM and amplitude. Peaks II and III are attributed to the coupling between different plasmonic modes, of which resonance wavelengths are almost unaffected by the increase in h. Therefore, EOT peak positions and intensity can be simultaneously selected by adjusting the thickness of Au film.

**Figure 6:**
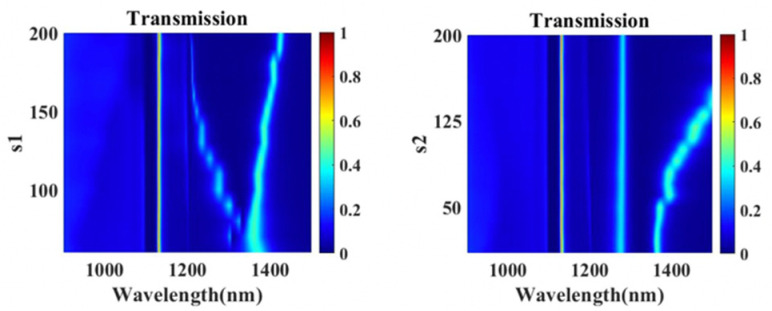
Simulated transmission spectra of the composite nanocavities metasurface with different S1 and S2.

The transmission spectrums of the composite nanocavities metasurface with different geometrical parameters S1 and S2 of vertical cavities are numerically investigated. As shown in Figure 6, transmission peak I remains almost unchanged, while peaks II and III are all shifted as S1 and S2 increase. S1 and S2 influence the coupling between plasmonic resonance in two vertical cavities, resulting in a change of the resonance wavelength of peaks II and III. Distance between different vertical cavities is utilized to control the spacing and position of the transmission peaks of the metasurface filter.

**Figure 7:**
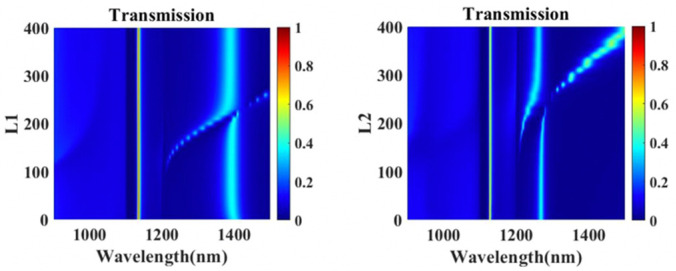
Simulated transmission spectra of the composite nanocavities metasurface with different lengths of vertical nanocavities L1 and L2.

The effect of lengths of the vertical nanocavities of the metasurface is also investigated. Transmission peak II is red-shifted with peaks I and III stay almost unchanged with L1 increasing from 0 to 400 nm in Figure 7. Similarly, an increase in L2 only causes the redshift of peak III. The length of a single vertical cavity only influences the EOT mode associated with itself and has no impact on other EOT peaks. Therefore, the EOT peaks II and III related to hybridization modes are able to be adjusted separately by lengths of the vertical nanocavities.

**Figure 8:**
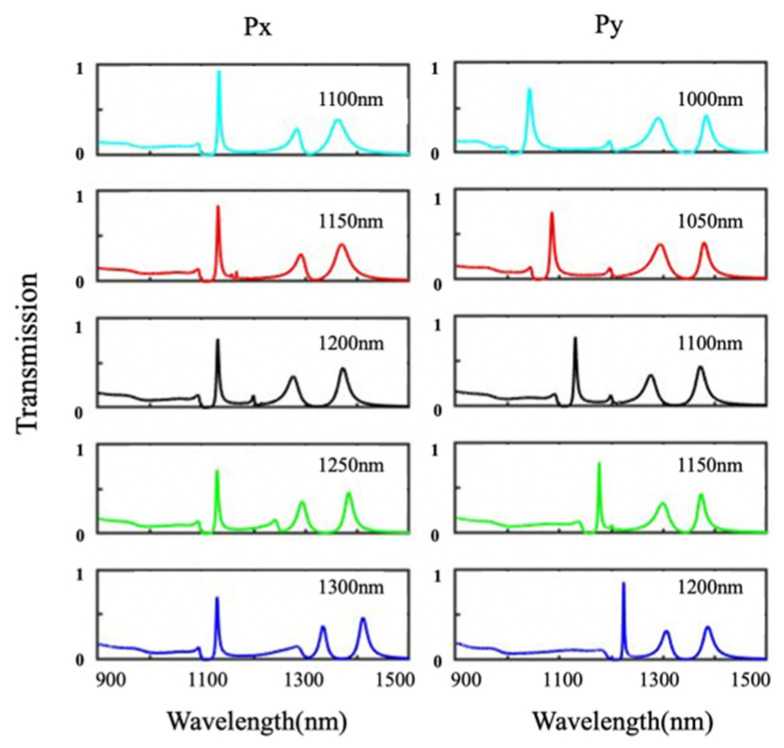
Simulated transmission spectra of the composite nanocavities metasurface with different periods Px and Py.

The effect of periods of the composite nanocavities metasurface on the transmission spectrum is also numerically calculated by the FDTD solution. When lateral period Px increases from 1100 to 1300 nm, peak I presents a slightly blue shift, and peaks II and III are red-shifted, as shown in Figure 8. With vertical period Py increasing from 1000 to 1250 nm, peak I has an obvious red shift, while peaks II and III are almost unchanged. The interaction of vertical cavities in adjacent periods is mainly influenced by Px, while that of the horizontal cavity is sensitive to Py, resulting in peak I and peaks II/III modulated by Py and Px, respectively. The spectral positions of EOT peak I and peaks II/III are independently tuned by the periods of metasurface in different directions.

**Figure 9:**
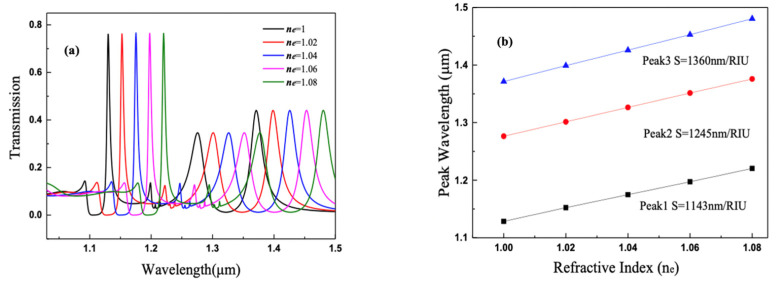
(**a**) Transmission spectra of composite cavities metasurface with different environmental refractive index n_e_. (**b**) The wavelength of three transmission peaks versus the refractive index n_e_.

The spectra response of environmental refractive index of composite nanocavities metasurface is also simulated by FDTD. As the environment refractive index n_e_ increases, the EOT peaks I, II, and III on transmission spectrum influenced by surface plasmon resonance are all red-shifted (shown in Figure 9a), and the refractive index sensitivity S (δλ/δn) are 1143, 1245, and 1360 nm/RIU (shown in Figure 9b), respectively. The transmission peaks of the composite nanocavities metasurface have quite high refractive index sensitivity. As a near-infrared multiple-channel filter, which is sensitive to the surrounding environment, it offers a feasible way to realize an environment-refractive tunable filter and provides the potential to be the integration component in nano-sensing.

**Figure 10:**
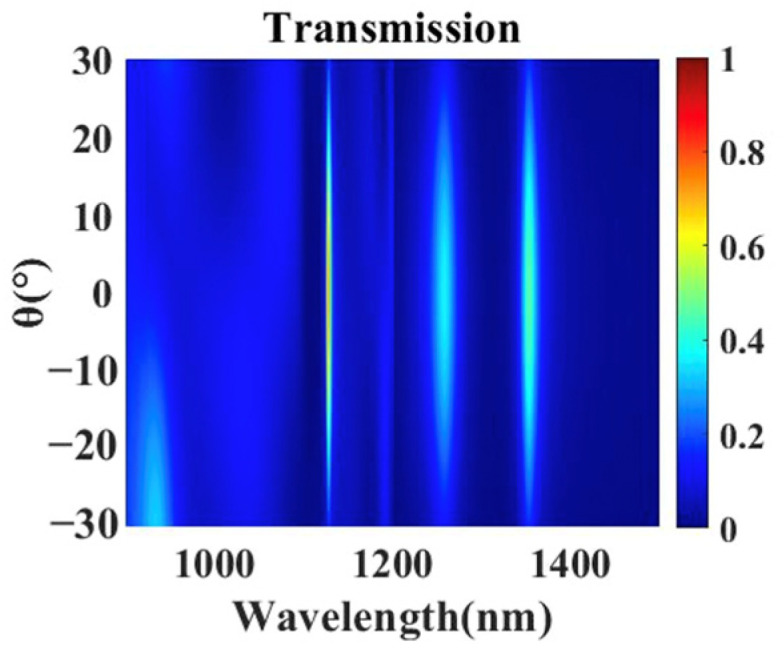
Simulated transmission spectra of the composite nanocavities metasurface with different incident angles θ (θ represents incident angle, and θ = 0 degree represents normal incidence).

The impact of incident angle on transmission spectrum is simulated as shown in Figure 10. Although transmission intensities of three peaks change simultaneously when the incident angle is varied, the wavelength positions do not shift. When the incident angle variation from the normal incidence is over ±10 degree, the impact on transmission intensity increased.

### 3.2. Experimental Results

**Figure 11:**
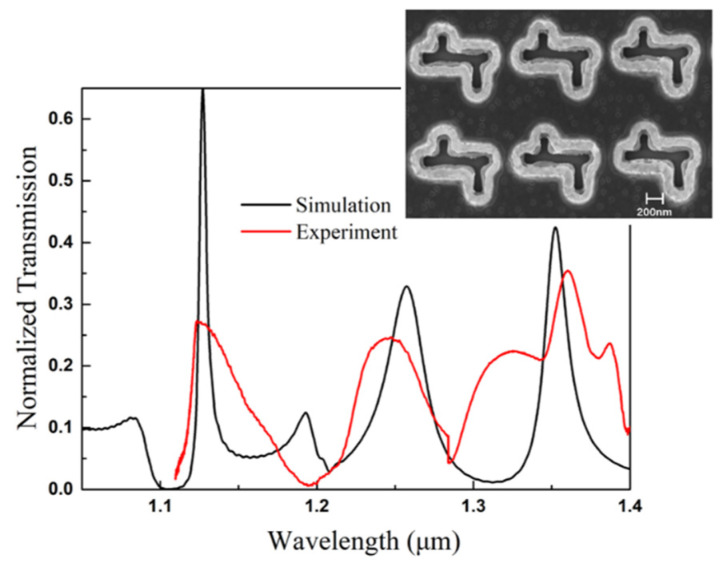
SEM images and the transmission spectrum of the fabricated composite nanocavities metasurface.

A plasmonic infrared multiple-channel filter based on the composite nanocavities metasurface was fabricated. The 225 nm-thick gold film was deposited on the SiO_2_ substrate by the Denton Electron Beam evaporator. The composite nanocavities metasurface was then fabricated on the gold film by Electron-beam lithography and Ion Beam Etching technique. The SEM image of the fabricated structure is shown in Figure 11. The geometrical parameters were measured in the SEM image: the length of the horizontal cavity is L1 = 802 nm, the lengths of vertical cavities are L2 = 204 nm and L3 = 301 nm, the horizontal and vertical periods are Px = 1187 nm and Py = 1091 nm, respectively. The size of the fabricated area is 1.2 mm × 1.1 mm. The transmission spectrum of the fabricated metasurface is detected by a Fourier-transform infrared spectrometer with a polarized source, which is shown as a red line in Figure 11. Transmission peaks at wavelengths 1128 nm, 1245 nm, and 1362 nm of the measured spectrum exhibit good accordance in spectral tendency and positions with the simulation result (black line in Figure 11). However, the narrow FWHW and high transmittance of the metasurface in the simulation were not entirely presented in the experiment.

Several possible reasons of difference between simulation and experimental result are analyzed: imprecision in the EBL process leads to a loss of fidelity; the IBE process may cause damage on the Au surface; the dielectric parameter of Au in the FDTD simulation is set as Palik model, which has a minor difference with that of experiment [30]; the ohmic loss within the Au film, etc. Especially, the transmittance of the first peak in the simulation was not entirely presented in the experiment, for the narrow FWHW making it more susceptible to facing the experiment imperfection.

For future research, a dielectric parameter of Au in the simulation could be set from experimental determination rather than the Palik model to decrease the difference between simulation and experimental results. The fabrication process might be improved by using other alternative techniques such as FIB with implantation of Ga+ [31] to increase the geometrical accuracy of the fabricated metasurface.

The composite nanocavities metasurface with a brief single-layer fabrication process achieved the multi-peak EOT phenomenon in the near-infrared region, which implements the multi-frequency selective filtering at the nanoscale.

## 4. Conclusions

In conclusion, a plasmonic infrared multiple-channel filter based on a gold periodic composite nanocavities metasurface is numerically analyzed and experimentally demonstrated. The multi-peak extraordinary optical transmission phenomenon of the metasurface is generated from the coupling between different plasmonic modes in composite cavities, processing multiple-channel filtering at wavelengths 1126 nm, 1255 nm, and 1350 nm in simulation. Surface plasmon resonances in composite cavities are affected by geometrical parameters of the cavities, periods of the metasurface, and environmental refractive index, which are utilized to tune frequency positions, spacing, and intensity of EOT peaks. The plasmonic composite nanocavities metasurface was fabricated by Electron-Beam Lithography and Ion Beam Etching technique. The experimentally measured spectrum of the fabricated metasurface by a Fourier-transform infrared spectrometer obtained three transmission peaks, which implemented multi-channel filtering at the nanoscale in the near-infrared region. The multi-frequency selective ability and brief single-layer fabrication of the proposed plasmonic near-infrared filter are essential for future spatial applications in nano-optical systems such as biosensing and detecting.

## Data Availability

Data is contained within the article.
